# The Newest Mania: Seeing Disease Mongering Everywhere

**DOI:** 10.1371/journal.pmed.0030319

**Published:** 2006-07-25

**Authors:** S. Nassir Ghaemi

**Affiliations:** **1**Emory UniversityAtlanta, GeorgiaUnited States of America

I feel compelled to comment on your article on bipolar disorder by my friend and colleague David Healy [ 1]. I respect Dr. Healy both as a historian of psychopharmacology and psychiatry and as a psychopharmacology researcher. I have been impressed by his historical scholarship over the years in bringing out the economic and social aspects of the rise of psychopharmacology. I think his specific critiques about the likely overuse of antidepressants in the West in recent years, as well as the influence of the pharmaceutical industry, have been valid in many respects. I also find the *PLoS Medicine'*s April 2006 series of articles on disease mongering not unconvincing, especially as it relates to new potential diagnoses like adult attention deficit hyperactivity disorder. Yet I must take exception to the inclusion of bipolar disorder with such newfangled entities.

Mania and melancholia have been well described since antiquity, and the current notions about the diagnosis of bipolar disorder (even the broader notions of the “bipolar spectrum”) are fully present in the writings of Esquirol and Kraepelin. It seems highly unlikely that they were markedly influenced by the pharmaceutical industry. To accept the drift of this collection of articles, one would have to suppose that Arataeus of Cappadocia was heavily influenced by pharmaceutical marketing in the 1st century a.d.

Of course, the possibility of overdiagnosis of bipolar disorder exists, often influenced by the pharmaceutical industry, but this in no way means that the diagnosis itself is invalid, nor does it counteract the much larger empirical evidence that bipolar disorder has been highly underdiagnosed (rather than the minimal empirical evidence that it is overdiagnosed) in the antidepressant era [ 2]. Dr. Healy seems to emphasize the issue in children, where indeed more uncertainty exists, but the overall impression of the article does not do justice to the reality that this illness has a long history of description and much more evidence of nosological validity (based on description, genetics, course, and biological data) [ 3] than such newcomers as adult attention deficit hyperactivity disorder and restless legs syndrome. Perhaps we should be on the lookout for the newest mania: seeing disease mongering everywhere.
